# Utility of CT Scan in Detecting Bladder Involvement Among Patients With Cervical Carcinoma

**DOI:** 10.7759/cureus.53670

**Published:** 2024-02-05

**Authors:** Rajni Agrawal, Ritika Agarwal

**Affiliations:** 1 Obstetrics and Gynecology, Venkateshwara Institute of Medical Science, Rajabpur, IND

**Keywords:** cystoscopy, ct scan, bladder, figo staging, cervical cancer

## Abstract

Background

Cervical cancer is a widespread health issue in India, particularly affecting women as the second most common cancer. The burden of cervical cancer in the country necessitates accurate staging for treatment optimization. The revised International Federation of Gynecology and Obstetrics (FIGO) staging system is vital for this purpose, emphasizing the extent of parametrial and pelvic sidewall involvement. Cervical cancer's propensity to infiltrate neighboring pelvic organs, including the bladder, necessitates precise staging. In India, traditional methods like cystoscopy have been relied upon, but they have limitations. Recent advancements in medical imaging, notably the increased use of computed tomography (CT) scans, provide a non-invasive alternative for staging and evaluating bladder involvement. This study aimed to evaluate the utility and accuracy of CT scans in assessing bladder involvement.

Methods

This cross-sectional study examined 127 newly diagnosed cervical carcinoma cases in women over a two-year period from August 2021 to July 2023. Patients underwent CT scans (plain) and cystoscopy, and bladder involvement was determined following the revised FIGO staging. Data collected comprised patient demographics, medical history, clinical symptoms, and FIGO staging. Cystoscopy was performed using an Olympus CYF-5 flexible cystoscope, and CT scans utilized a 64-slice multidetector CT scanner. Radiological reports detailed primary tumor characteristics and proximity to the bladder. Statistical analysis encompassed descriptive statistics, and calculation of sensitivity, specificity, positive predictive value, and negative predictive value for CT scans in comparison to cystoscopy. Statistical significance was considered at p < 0.05.

Results

In our study, the mean participant age was 45.3 years, with 61.4% falling in the 40-60 years age group. Socioeconomic status (SES) varied, with 37.8% classified as low SES, 48.8% as middle SES, and 13.4% as high SES. Parity data showed that 76.4% had three or more pregnancies. Among presenting symptoms, abnormal vaginal bleeding (65.4%) was the most prevalent, and squamous cell carcinoma (78.7%) was the predominant histological type. The prevalence of bladder involvement was 9.4% by cystoscopy and 30.7% by CT scans. CT scan demonstrated a high sensitivity (100%) but lower specificity (76.52%), with 78.80% overall accuracy.

Conclusion

A combined approach, using CT scans as a screening tool and cystoscopy as a confirmatory method, could provide the most comprehensive and reliable assessment of bladder involvement in cervical carcinoma patients, ultimately contributing to improved patient care and management.

## Introduction

Cervical cancer is a pervasive health concern in India, where it ranks as the second most common cancer among women. According to the GLOBOCAN database for the year 2020, India witnessed an alarming 122,844 new cases of cervical cancer, with an estimated 67,477 deaths attributed to this malignancy [1]. The burden of cervical cancer in India underscores the imperative need for accurate staging and diagnosis to optimize treatment strategies and improve patient outcomes.

The International Federation of Gynecology and Obstetrics (FIGO) staging system plays a pivotal role in guiding clinical decisions and treatment planning for cervical cancer patients in India. The revised FIGO staging system, which considers the extent of parametrial and pelvic sidewall involvement, has been instrumental in enhancing the precision of cervical cancer staging [2,3].

Cervical cancer is known for its propensity to infiltrate neighboring pelvic organs, with bladder involvement being a significant concern. This can substantially alter the treatment approach and necessitate a multidisciplinary management strategy [4]. In the Indian context, where a vast and diverse population has varying access to healthcare resources, precise staging is crucial for ensuring optimal therapeutic interventions [5].

Traditional methods for assessing bladder involvement, such as cystoscopy, have long been relied upon in India [5]. Cystoscopy, however, is not without its limitations, as it is an invasive procedure that can be uncomfortable for patients. Moreover, it may not always provide a comprehensive evaluation of bladder involvement, particularly in cases where tumor infiltration is not readily visible within the bladder lumen [6].

In recent years, India has witnessed notable advances in medical imaging, particularly the use of Computed Tomography (CT) scans [7]. These scans offer a non-invasive means of evaluating cervical cancer staging and the extent of bladder involvement. With a substantial increase in the availability and utilization of CT scans across India, this imaging modality holds promise as an alternative or complementary tool for assessing bladder involvement [7].

The necessity for a reliable and less invasive method to evaluate bladder involvement in Indian cervical cancer patients has led to the exploration of CT scans as an option in clinical practice [8]. The utility and accuracy of CT scans in determining bladder involvement within the context of the revised FIGO staging criteria are subjects of ongoing research and discussion [9-11].

The aim of this study was to evaluate and compare the findings of CT scans and cystoscopy in assessing bladder involvement in patients with carcinoma cervix according to the revised FIGO staging. By doing so, we intended to address the following objectives: Assess the sensitivity and specificity of CT scans in comparison to cystoscopy in detecting bladder involvement in cervical cancer; examine the concordance and discordance between these diagnostic methods in identifying bladder involvement; and evaluate the clinical implications of these findings, including their impact on treatment decisions and patient outcomes. This research will not only contribute to the ongoing effort to refine cervical cancer staging but also guide clinicians in choosing the most appropriate diagnostic approach, particularly in resource-constrained settings. Ultimately, this study aimed to enhance the accuracy of staging in cervical cancer and improve patient care and outcomes.

## Materials and methods

Study design and setting

This cross-sectional study was conducted at Venkateshwara Institute of Medical Science, Rajabpur, specializing in the Obstetrics and Gynecology Department. The study spanned a period of two years from August 2021 to July 2023.

Study population

The study included a cohort of patients newly diagnosed (histopathologically) with cervical carcinoma (including those who had not undergone any prior treatment) during a defined study period. Patients with contraindications for CT scans or cystoscopy, prior bladder surgery, or a history of other malignancies affecting the bladder were excluded.

Sample size calculation

The sample size was calculated based on an estimated prevalence of bladder involvement in cervical cancer patients according to Bhatla et al. [5]. With a desired level of significance (α) set at 0.05 and a power (1-β) of 0.80, the minimum sample size required was calculated as 87 using the formula for sample size calculation in a cross-sectional study, where the estimated prevalence of bladder involvement is 6%. However, during a defined period, a total of 127 newly diagnosed (histopathologically) cervical carcinomas were presented, so the total sample size of our study was 127.

Data collection

A predesigned proforma was used to collect patients' details such as patient demographics including age, and other relevant characteristics. The medical history of each patient was obtained through a combination of electronic health record (EHR) review and patient interviews. Past medical conditions, previous cancer diagnoses, surgical history, and any history of pelvic radiation therapy were documented. The presenting symptoms and clinical signs at the time of diagnosis were carefully documented. These clinical presentations included abnormal vaginal bleeding, pelvic pain, urinary symptoms, or other relevant complaints that might suggest cervical cancer.

The FIGO staging system is a fundamental aspect of cervical cancer assessment and management, as it provides a standardized framework for categorizing the extent of disease. In this study, FIGO staging was applied consistently to all patients to determine the stage of cervical cancer. The revised FIGO staging system was utilized to ensure uniformity and accuracy in assessing the extent of the disease. Trained gynecologists performed thorough pelvic examinations for all patients as part of the staging process. The primary tumor was carefully assessed in terms of size, location, and extent. This examination provided initial insights into the extent of the cervical tumor. The gynecologists also assessed for parametrial involvement, a critical component of FIGO staging. Parametrial involvement signifies that the cancer has spread to the supporting tissues around the cervix and can significantly impact the stage of the disease, and the presence of regional lymphadenopathy in the pelvis was examined during the pelvic assessment. Lymph node involvement is an important criterion in determining the stage of cervical cancer according to FIGO guidelines.

Cystoscopy was performed using an Olympus CYF-5 flexible cystoscope. This equipment is equipped with a light source and an optical system to provide clear visualization of the interior of the bladder. Prior to the cystoscopy, patients were prepared according to established protocols. This typically includes cleaning the genital and perineal areas to reduce the risk of infection, ensuring the patient's comfort, and providing necessary anesthesia or local numbing agents. Cystoscopy procedures were performed by experienced urologists who are skilled in conducting this procedure. The expertise of the urologists is critical for accurate visualization, precise assessment, and, if necessary, the collection of biopsy samples. Cystoscopy provides a direct and real-time view of the interior of the bladder. The cystoscope is inserted into the patient's urethra, advanced into the bladder, and manipulated to examine all areas of the bladder, including the bladder wall, the trigone (the triangular area of the bladder), and any other relevant regions. The urologist carefully examines the bladder mucosa, looking for any visible signs of tumor invasion, such as nodules, ulceration, or changes in the bladder's normal architecture. Any suspicious areas are documented and, if necessary, biopsy samples are obtained for histological confirmation. Cystoscopy is instrumental in assessing the extent of bladder involvement. Urologists evaluate the depth of tumor infiltration into the bladder wall, as well as its proximity to critical structures and anatomical landmarks. The urologists use the cystoscope to gauge the extent of tumor involvement, providing critical data for determining the patient's stage according to the FIGO staging system.

The CT scans were conducted using a 64-slice multidetector CT scanner (Siemens SOMATOM Definition AS+) with high-resolution imaging parameters. Following the CT scans, radiological reports were generated by specialist radiologists who were experienced in interpreting oncological imaging. These reports detailed the findings of the CT scans, including the size and location of the primary cervical tumor, its proximity to the bladder, and any potential bladder wall invasion. These findings were categorized and graded according to the revised FIGO staging system. CT scans also provided valuable information regarding the presence of regional lymph node enlargement. This is an important aspect of cervical cancer staging and is a criterion within the FIGO staging system. The CT scans were instrumental in determining the extent of bladder involvement by evaluating the proximity of the cervical tumor to the bladder, any visible signs of infiltration, and any associated anatomical changes in the bladder wall.

In cases where clinical and radiological findings were inconclusive, or there was a need to confirm the histological diagnosis and stage, a biopsy of lesions was performed. These biopsies provided definitive information for accurate staging.

The revised FIGO staging system divides cervical cancer into several categories based on the extent of tumor involvement. These categories range from Stage 0 (in-situ carcinoma) to Stage IV (distant metastasis). Each stage is defined by specific criteria, including the size of the primary tumor, parametrial involvement, lymph node status, and the presence of distant metastasis. Staging according to FIGO guidelines is a critical step in the diagnostic process, as it not only helps in determining prognosis but also guides treatment decisions. The choice of treatment, which can include surgery, radiation therapy, or chemotherapy, is often stage-dependent. To ensure consistency and accuracy in applying FIGO staging, the clinical and radiological assessments were performed by experienced medical professionals who were well-versed in the FIGO guidelines. This minimizes interobserver variability and enhances the reliability of the staging process. Regular meetings and consensus discussions among the medical team were conducted to address any discrepancies or challenging cases to arrive at a consensus for the most appropriate stage.

Statistical analysis

Data were analyzed using SPSS 20.0 (IBM Corp., Armonk, NY), with descriptive statistics applied for patient demographics, clinical characteristics, and the prevalence of bladder involvement. Sensitivity, specificity, positive predictive value, and negative predictive value were calculated for CT scans, considering cystoscopy as the reference standard. A p-value of <0.05 was considered statistically significant.

Ethical considerations

This study was conducted in accordance with the ethical principles outlined in the Declaration of Helsinki. Ethical approval was obtained from the Institutional Ethics and Review Board of Venkateshwara Institute of Medical Science, Rajabpur, India (approval number: VIMS/IERB/2021/05/0188). Informed consent was obtained from all participants, and their privacy and confidentiality were strictly maintained.

## Results

In our study, the mean age of the participants was 45.3 years. Age distribution revealed that the majority of patients fell into the 40-60 years age group, constituting 61.4% of the cohort, while those aged below 40 years and above 60 years accounted for 28.3% and 10.3%, respectively. Socioeconomic status (SES) demonstrated a diverse distribution, with 48 (37.8%) participants categorized as having a low SES, 62 (48.8%) with a middle SES, and 17 (13.4%) belonging to the high SES category. Parity data revealed that 30 (23.6%) patients were parity <3, while the majority, 97 (76.4%), had three or more pregnancies. Menopausal status indicated that 55 (43.7%) participants were premenopausal, and 72 (56.3%) were postmenopausal (Table 1).

**Table 1:** Demographic characteristics of study participants (N=127).

Characteristic	Number (%)/ Mean ± SD
Age (years), Mean ± SD	45.3 ± 6.2
Age Groups (years)	
<40 years	36 (28.3%)
40-60 years	78 (61.4%)
>60 years	13 (10.3%)
SES (Socioeconomic Status)	
Low	48 (37.8%)
Middle	62 (48.8%)
High	17 (13.4%)
Parity	
<3	30 (23.6%)
3 or more	97 (76.4%)
Menopausal Status	
Premenopausal	55 (43.7%)
Postmenopausal	72 (56.3%)

In our study, among presenting symptoms, abnormal vaginal bleeding was the most prevalent, affecting 65.4% of patients, followed by pelvic pain (37.0%), urinary symptoms (21.3%), and other relevant complaints (12.6%). In terms of medical history, 8.7% of patients had a prior history of cancer, 14.2% had undergone previous surgeries, and 6.3% had received pelvic radiation therapy. Clinical findings indicated a mean tumor size of 4.7 cm with a standard deviation of 1.2. Tumor location revealed that the anterior wall was the most commonly affected site (41.7%), followed by the posterior wall (32.3%) and the lateral wall (26.0%). FIGO staging demonstrated a varied distribution, with 15.7% of patients diagnosed at Stage I (early stage), 42.5% at Stage II (locally advanced), 31.5% at Stage III (advanced), and 10.3% at Stage IV (metastatic). Histopathological analysis revealed squamous cell carcinoma as the predominant type, accounting for 78.7% of cases, followed by adenocarcinoma (17.3%) and other histological types (4.0%). Histological grading indicated that 23.6% of tumors were well-differentiated, 51.2% were moderately differentiated, and 25.2% were poorly differentiated (Table 2).

**Table 2:** Clinical characteristics of cervical carcinoma among study participants (N=127).

Characteristic	Number (%)/ Mean ± SD
Presenting Symptoms	
Abnormal Vaginal Bleeding	83 (65.4%)
Pelvic Pain	47 (37.0%)
Urinary Symptoms	27 (21.3%)
Other Relevant Complaints	16 (12.6%)
Medical History	
Previous Cancer	11 (8.7%)
Surgical History	18 (14.2%)
Pelvic Radiation	8 (6.3%)
Clinical Findings	
Tumor Size (cm), Mean ± SD	4.7 ± 1.2
Tumor Location	
Anterior Wall	53 (41.7%)
Posterior Wall	41 (32.3%)
Lateral Wall	33 (26.0%)
FIGO Stage	
Stage I (Early stage)	20 (15.7%)
Stage II (Locally advanced)	54 (42.5%)
Stage III (Advanced)	40 (31.5%)
Stage IV (Metastatic)	13 (10.3%)
Histopathology	
Squamous Cell Carcinoma	100 (78.7%)
Adenocarcinoma	22 (17.3%)
Other Histological Types	5 (4.0%)
Histological Grade	
Well Differentiated	30 (23.6%)
Moderately Differentiated	65 (51.2%)
Poorly Differentiated	32 (25.2%)

Bladder involvement in patients with cervical carcinoma is a critical aspect of disease assessment, and the study findings shed light on this crucial dimension. Among the 127 patients included in the study, cystoscopy and CT scans were employed to evaluate bladder involvement, and the prevalence of bladder involvement was 9.4% on cystoscopy (12/127), and 30.7% on CT scan (39/127). In the CT Scan Group, which encompassed 39 patients, the findings revealed a spectrum of FIGO stages. Notably, a minority of patients were diagnosed at Stage I, constituting 2.6% of the group, while Stage II patients accounted for 10.3%. The majority, a significant 59.0% of patients, were classified as Stage III, indicating advanced disease. Furthermore, 28.2% of patients in this group were categorized as Stage IV, signifying metastatic disease. In contrast, the Cystoscopy Group, comprising 12 patients, exhibited a slightly different distribution. While there were no Stage I patients, indicating that cystoscopy may not be as sensitive in detecting early-stage disease, Stage II was represented by 8.3% of the group. Impressively, Stage III patients constituted the majority, with 66.7%, showcasing the utility of cystoscopy in identifying advanced stages. Additionally, Stage IV patients represented 25.0% of this group (Table 3).

**Table 3:** Prevalence of bladder involvement and FIGO staging. FIGO - International Federation of Gynecology and Obstetrics

FIGO stage	CT Scan Group (n=39) Number (%)	Cystoscopy Group (n=12) Number (%)
FIGO Stage I (n=20)	1 (2.6%)	0 (0.0%)
FIGO Stage II (n=40)	4 (10.3%)	1 (8.3%)
FIGO Stage III (n=54)	23 (59.0%)	8 (66.7%)
FIGO Stage IV (n=13)	11 (28.2%)	3 (25.0%)

In our study, the mean tumor size within the involved bladder was calculated at 4.8 cm, indicating the size of the malignancies within the bladder. Tumor location analysis revealed the distribution of bladder involvement, with 33.3% affecting the anterior wall, 25.0% impacting the posterior wall, and 41.7% localized in the lateral wall. This diverse distribution underscores the variable sites at which bladder involvement occurs in cervical cancer patients. Assessing the extent of bladder involvement, it was found that 16.7% of cases were classified as superficial, 50.0% as submucosal, and 33.3% as muscle invasive, providing a comprehensive insight into the depth of tumor infiltration into the bladder wall. Lymph node involvement is a crucial aspect of cervical cancer staging, and in this subgroup, 25.0% of patients exhibited lymph node involvement, while the majority, 75.0%, had no such involvement, indicating regional variations in disease progression. It is noteworthy that in all cases of bladder involvement, histological confirmation was achieved, highlighting the importance of precise histopathological assessment in cases where bladder involvement is suspected (Table 4).

**Table 4:** Characteristics of bladder involvement on cystoscopy in cervical cancer patients (N=12).

Characteristic of Bladder involvement	Number (%)/ Mean ± SD
Tumor Size (cm), Mean ± SD	4.8 ± 1.2
Tumor Location	
Anterior Wall	4 (33.3%)
Posterior Wall	3 (25.0%)
Lateral Wall	5 (41.7%)
Tumor Extent	
Superficial	2 (16.7%)
Submucosal	6 (50.0%)
Muscle Invasive	4 (33.3%)
Lymph Node Involvement	
Present	3 (25.0%)
Absent	9 (75.0%)
Histological Confirmation of Bladder Involvement	12 (100.0%)

In this comparison, CT scans, when considered in relation to cystoscopy, demonstrated excellent sensitivity of 100%, signifying their efficacy in correctly identifying cases of bladder involvement. However, the specificity of 76.52% indicates a moderate ability to accurately determine cases without bladder involvement. The positive likelihood ratio of 4.26 suggests a substantial increase in the likelihood of true bladder involvement when CT scans are positive. The positive predictive value of 31.39% indicates the probability that a positive CT scan result accurately represents bladder involvement. The high negative predictive value of 100% underscores the reliability of CT scans in excluding bladder involvement. The overall accuracy of 78.80% affirms the effectiveness of CT scans in assessing bladder involvement in cervical carcinoma, in comparison to cystoscopy (Table 5).

**Table 5:** Comparison of CT scan and cystoscopy findings for bladder involvement.

Comparison	Cystoscopy Positive (Number)		Cystoscopy Negative (Number)		Total (Number)
CT Scan Positive	12		27		39
CT Scan Negative	0		88		88
Total (Number)	12		115		127
Statistic		Value		95% CI	
Sensitivity		100.00%		73.54% to 100.00%	
Specificity		76.52%		67.71% to 83.92%	
Positive Likelihood Ratio		4.26		3.06 to 5.92	
Positive Predictive Value		31.39%		24.75% to 38.89%	
Negative Predictive Value		100.00%		95.89% to 100.00%	
Accuracy		78.80%		70.66% to 85.55%	

## Discussion

Cervical carcinoma, a significant global health concern, demands precise staging for effective management and treatment planning. Accurate assessment of bladder involvement is pivotal, as it impacts both prognosis and treatment decisions. With developments in imaging techniques, cross-sectional imaging modalities like CT and magnetic resonance imaging (MRI) are frequently used. In contrast, conventional imaging methods such as intravenous urography (IVU) and barium enema have almost become obsolete in the staging workup of cervical carcinoma. MRI is the imaging modality of choice for local staging of cervical carcinoma. Positron emission tomography-CT (PET-CT) is not suitable for tumor staging in early cervical cancers; however, it is useful and more accurate than MRI for the detection of lymph nodes and distant metastases and, hence, is desirable in locally advanced cervical cancer. MRI is superior to CT in the depiction of the primary tumor. However, in developing countries where the burden of cervical cancer is high, CT is the preferred modality, as it is more widely available and less expensive [12-14].

The assessment of bladder involvement in cervical cancer patients via cystoscopy revealed valuable insights into the extent of this critical aspect of the disease. In our study, the prevalence of bladder involvement via cystoscopy was 9.4% (12/127). Varying prevalence rates of bladder involvement were observed in various studies. Prasad et al. reported a prevalence of 4%, Jeong et al. found 4.23%, Sharma et al. detected bladder involvement in 5.59%, Chung et al. reported 2.7%, Sundborg et al. found 6.1%, and Liang et al. reported 8% prevalence of bladder involvement through cystoscopy [15-20]. Assessing bladder involvement through CT scans provided further insights into the extent of the disease in cervical cancer patients. In our study, the prevalence of bladder involvement via CT scan was 30.7% (39/127). Varying prevalence rates of bladder involvement were observed in various studies. Prasad et al. identified bladder involvement in 26%, Jeong et al. reported 4.91%, Sharma et al. revealed bladder involvement in 13.81%, Chung et al. found a prevalence of 2.7%, Sundborg et al. identified bladder involvement in 10.2%, and Liang et al. reported a prevalence of 10% for bladder involvement as detected by CT scan [15-20].

The exceptional sensitivity of CT scans, reaching 100%, emphasizes their proficiency in correctly identifying cases with bladder involvement. This aligns with the non-invasive nature of CT scans, making them a valuable tool in screening for bladder-related concerns in cervical carcinoma patients. However, the specificity of 76.52% suggests a moderate precision in discerning cases without bladder involvement, indicating the importance of corroborative diagnostic approaches. The positive likelihood ratio of 4.26 underscores the substantial increase in the likelihood of true bladder involvement when CT scans yield positive results. While the positive predictive value of 31.39% implies a relatively low probability that a positive CT scan accurately represents bladder involvement, the high negative predictive value of 100% reaffirms the reliability of CT scans in excluding bladder involvement. The overall accuracy of 78.80% emphasizes the effectiveness of CT scans as a valuable diagnostic tool for assessing bladder involvement in cervical carcinoma, especially when considering the limitations of cystoscopy [10,11].

In the study conducted by Sundborg et al., the CT scan exhibited an exceptional sensitivity of 100% and a high specificity of 96%. While it had a moderate PPV of 60%, the NPV was 100%, indicating that when a CT scan indicated the absence of bladder involvement, it was highly reliable. The overall accuracy was 96% [19]. Liang et al. reported a study, where a CT scan demonstrated a sensitivity of 100% and an impressive specificity of 98%. The PPV was 80%, and the NPV was 100%, further emphasizing the reliability of CT scan. The overall accuracy was 98% [20]. Massad et al. conducted a study and their findings indicated that CT scan had a sensitivity of 100%, a specificity of 95%, and an NPV of 91%. These results suggest a limitation in the specificity of CT scans in this particular study, potentially leading to false-positive results [21]. Chung et al., in a study, reported a sensitivity of 100% and a specificity of 98% for CT scans. The PPV was 57%, and the NPV was 100%, underlining the utility of CT scans in accurately diagnosing bladder involvement. The overall accuracy was 98% [18]. Sharma et al. conducted a study and reported that a CT scan demonstrated a sensitivity of 100% and a high specificity of 92%. However, the PPV was 40%, indicating that while a CT scan is reliable in identifying cases without bladder involvement, it may over-diagnose bladder involvement. The NPV was 100%, and the overall accuracy was 92% [17]. In the study by Jeong et al., CT scans exhibited a sensitivity of 68.2% and a high specificity of 96.4%. The PPV was 51.7%, and the NPV was 100%, indicating that while a CT scan is highly reliable in confirming cases without bladder involvement, it may have limitations in correctly identifying bladder involvement. The overall accuracy was 98% [16]. Finally, Prasad et al. conducted a study, and their findings showed that CT scans had a sensitivity of 100% and a specificity of 77%. The PPV in this study was 15.38%, while the NPV was 100%, indicating that while a CT scan is reliable in ruling out bladder involvement, its reliability in diagnosing bladder involvement might vary. The overall accuracy was 78% [15].

In the study by Ozsarlak et al., the overall accuracy of CT was reported to be 47% [22]. However, Hancke et al. reported that clinical examination was more accurate than CT [23]. Furthermore, a previous study conducted by Whitley et al. also demonstrated poor sensitivity of CT in diagnosing pelvic wall invasion [24]. These findings emphasize the limitations of CT in certain aspects of disease diagnosis and staging.

The diagnostic efficacy of these methods becomes even more apparent when considering the implications for patient management. Cystoscopy may serve as a valuable tool in confirming bladder involvement, guiding surgeons during procedures, and reducing the risk of incomplete tumor resections. CT scans, on the other hand, provide an excellent overview of the pelvic region, allowing for the evaluation of local tumor extent and the assessment of lymph node involvement, aiding in accurate FIGO staging.

In this context, a combined approach could offer the most comprehensive assessment. CT scan, with its high sensitivity, could be employed as a confirmatory tool when clinical or radiological findings are inconclusive. The specificity of CT scans may make them suitable as a screening tool, identifying patients for further, more targeted evaluation via cystoscopy. Therefore, an integrated approach using both methods could potentially offer the most robust diagnostic strategy, ensuring the highest level of accuracy and the avoidance of misdiagnoses [25,26].

Limitations

Despite these insights, our study has limitations. The sample size, while sufficient for the objectives of our study, is relatively small. A larger and more diverse patient cohort could provide additional insights into the diagnostic accuracy of these methods. Furthermore, the study's design was cross-sectional, and longitudinal data would be valuable in assessing the long-term impact of different diagnostic strategies on patient outcomes.

## Conclusions

In conclusion, the accurate assessment of bladder involvement in cervical carcinoma patients is of paramount importance in determining treatment options and prognostic evaluation. CT scan, with its exceptional specificity, is highly reliable in confirming the presence of bladder involvement. The high sensitivity and NPV of CT in determining urinary bladder invasion make CT an effective preliminary screening modality and cystoscopy can be done in selective patients. A combined approach, using CT scans as a screening tool and cystoscopy as a confirmatory method, could provide the most comprehensive and reliable assessment of bladder involvement in cervical carcinoma patients, ultimately contributing to improved patient care and management. Further research and larger-scale studies are warranted to confirm and expand upon these findings.
